# A novel combination of the Arndt endobronchial blocker and the laryngeal mask airway ProSeal™ provides one-lung ventilation for thoracic surgery

**DOI:** 10.3892/etm.2014.1966

**Published:** 2014-09-15

**Authors:** QIONG LI, PEIYING LI, JIANGHUI XU, HUAHUA GU, QINYUN MA, LIEWEN PANG, WEIMIN LIANG

**Affiliations:** 1 Department of Anesthesiology, Huashan Hospital, Fudan University, Shanghai 200042, P.R. China; 2 Department of Thoracic Surgery, Huashan Hospital, Fudan University, Shanghai 200042, P.R. China

**Keywords:** one-lung ventilation, laryngeal mask airway, Arndt endobronchial blocker

## Abstract

In this study, the feasibility and performance of the combination of the Arndt endobronchial blocker and the laryngeal mask airway (LMA) ProSeal™ in airway establishment, ventilation, oxygenation and lung isolation was evaluated. Fifty-five patients undergoing general anesthesia for elective thoracic surgeries were randomly allocated to group Arndt (n=26) or group double-lumen tube (DLT; n=29). Data concerning post-operative airway morbidity, ease of insertion, hemodynamics, lung collapse, ventilators, oxygenation and ventilation were collected for analysis. Compared with group DLT, group Arndt showed a significantly attenuated hemodynamic response to intubation (blood pressure, 149±31 vs. 115±16 mmHg; heart rate, 86±15 vs. 68±15 bpm), less severe injuries to the bronchus (injury score, 1.4±0.2 vs. 0.4±0.1) and vocal cords (injury score, 1.3±0.2 vs. 0.6±0.1), and lower incidences of post-operative sore throat and hoarseness. Furthermore, the novel combination of the Arndt and the LMA ProSeal showed similar ease of airway establishment, comparable ventilation and oxygenation performance, and an analogous lung isolation effect to DLT. The novel combined use of the Arndt endobronchial blocker and the LMA ProSeal can serve as a promising alternative for thoracic procedures requiring one-lung ventilation. The less traumatic properties and equally ideal lung isolation are likely to promote its use in rapidly spreading minimally invasive thoracic surgeries.

## Introduction

The laryngeal mask airway (LMA) is a relatively new device that was brought into clinical practice in the 1980s. The LMA avoids undergoing laryngoscopy as well as all of its adverse effects. Additionally, it is less invasive to the respiratory tract and tracheal oedema, which may be caused by tracheal intubation, does not occur by using the LMA. Furthermore, it may be life saving in patients with malformations of the upper airway after a failure of tracheal intubation and mask ventilation. The laryngeal mask airway (LMA) is considered to be an extremely useful alternative to the endotracheal tube (ETT) in a number of clinical scenarios. Compared with conventional ETTs, the LMA causes less airway resistance, a decreased bronchoconstrictive reflex, less atelectasis and fewer pulmonary infections (1). However, the application of the LMA in thoracic anesthesia has been limited due to the requirement of one-lung ventilation (OLV), which appeared impossible for the LMA. Although reported as a useful adjunct in patients undergoing thoracotomy (2), the LMA alone has no possibility for OLV. In 1999, the Arndt endobronchial blocker, designed by Dr G.A. Arndt (3,4), emerged as a versatile alternative to double-lumen tubes (DLTs) to facilitate OLV in thoracic anesthesia (5–8).

Previously, the combination of the LMA and the Arndt endobronchial blocker was reported in a pediatric scoliosis case (9). We hypothesized that the combination could also be applied safely to thoracic cases, bringing the advantages of fewer airway injuries, fewer fluctuations in hemodynamics and fewer limitations for OLV, enabling use in patients with difficult airways and in pediatric patients.

In the present prospective pilot study, the feasibility of OLV provided by the combination of the Arndt endobronchial blocker and the LMA ProSeal™ was assessed, and comparisons of the post-operative airway morbidity and the respiratory and hemodynamic data regarding the two strategies of OLV were performed.

## Materials and methods

### Patient recruitment

The study was approved by the Human Ethics Committee of Huashan Hospital (Shanghai, China) and written informed consent was obtained from all the patients. Fifty-five adult patients (age, 20–70 years; American Society of Anesthesiologists physical status, I-III) who were undergoing day-case thoracic surgeries were entered into the study prospectively. All patients were randomly allocated to group Arndt (n=26) or group DLT (n=29). Exclusion criteria included an age of <20 years, pre-operative hoarseness, increased risk of aspiration, mouth opening of <2.5 cm and surgeries predicted to have long durations.

### Anesthesia procedure

Anesthetic monitoring, including electrocardiography, pulse oximetry, capnography and non-invasive blood pressure monitoring, was conducted prior to anesthesia induction and during the procedure. Prior to the induction of anesthesia, an arterial line was established for determining the baseline of arterial blood gas, blood pressure and heart rate. All patients were anesthetized by two chief residents who were experienced in the use of the LMA, the Arndt endobronchial blocker and DLT. General anesthesia was induced and maintained with target controlled infusion using a Graseby™ 3500 syringe pump (Smith Medical MD, Inc., St. Paul, MN, USA) of propofol at 4.0 μg/ml (plasma concentration) and continuous infusion of 3 μg/kg/h fentanyl and 0.8 mg/kg/h rocuronium. In group Arndt, the LMA ProSeal (LMA North America, Inc., San Diego, CA, USA) was placed and the patient was ventilated with pressure-controlled mode to achieve a tidal volume of 10 ml/kg (Primus Dräger, Lübeck, Germany). An Arndt endobronchial blocker (Cook Medical Critical Care, Bloomington, IN, USA) was subsequently placed and guided by a 3.4-mm fiberoptic scope (FOB; BF type 3 C40; Olympus, Tokyo, Japan). In group DLT, the patients were intubated with DLTs and were ventilated with the same ventilator regimen. Positioning was confirmed by the same FOB. The blood pressures were read from the arterial line and the heart rates were recorded following placement of the airway devices and cuff position confirmation. A manometer for LMA (Mallinckrodt, Griesheim, Germany) was used to measure and control the cuff pressure between 55 and 60 cmH_2_O. Peak airway pressure and compliance were measured using an S/5 Compact Anesthesia Monitor (Datex-Ohmeda, Madison, WI, USA) and recorded while the patient was in both the supine and the lateral decubitus positions. Subsequent to patients being positioned to the lateral decubitus, the cuff position of the endobronchial blocker or of the DLT’s endobronchial tube was checked again.

### Evaluation of lung isolation

The evaluation of lung collapse and surgery exposure was performed by a thoracic surgeon blinded to the group assignment. Collapse of the lung was assessed as follows: 1, spontaneous; 2, assisted with suction; 3, manual. The conditions of surgery were rated as follows: 1, excellent (complete collapse with perfect surgical exposure); 2, fair (total collapse, but with residual air remaining in the lung); 3, poor (no collapse or partial collapse with interference in surgical exposure) (10).

### Bronchoscopic examination

Upon completion of surgery, all patients underwent a bronchoscopic examination prior to emerging from anesthesia. Findings from the bronchoscopy and laryngoscopy were classified into bronchus, vocal cord and larynx injuries, and each injury class was scored as follows (10): 0, no changes; 1, redness; 2, edema; and 3, hematoma.

### Assessment of post-operative airway morbidity

For the first three days after surgery, post-operative airway morbidity was rated by the anesthesia resident blinded to the group assignment. The assessment was performed in accordance with that described in the study by Knoll *et al* (10).

### Statistical analysis

Patients were randomized using the sealed envelope system. All patients were stratified on gender and airway resistance information obtained from the pre-operative spirometry examination. Statistical analysis was performed using SPSS software version 14.0 (SPSS Inc., Chicago, IL, USA). Results were considered statistically significant when P<0.05. Data are expressed as the mean ± standard deviation. The independent samples t-test and Mann-Whitney U test were used for analyzing parametric and nonparametric data as appropriate.

## Results

Fifty-five patients were enrolled in this study. The two groups were comparable in terms of age, male/female ratio, height, weight, pre-operative spirometry results and pre-operative hemodynamic parameters (Table I). In the context of the types of surgical procedures and the establishment of airways, the two groups were comparable (Tables II and III).

**Figure 1:**
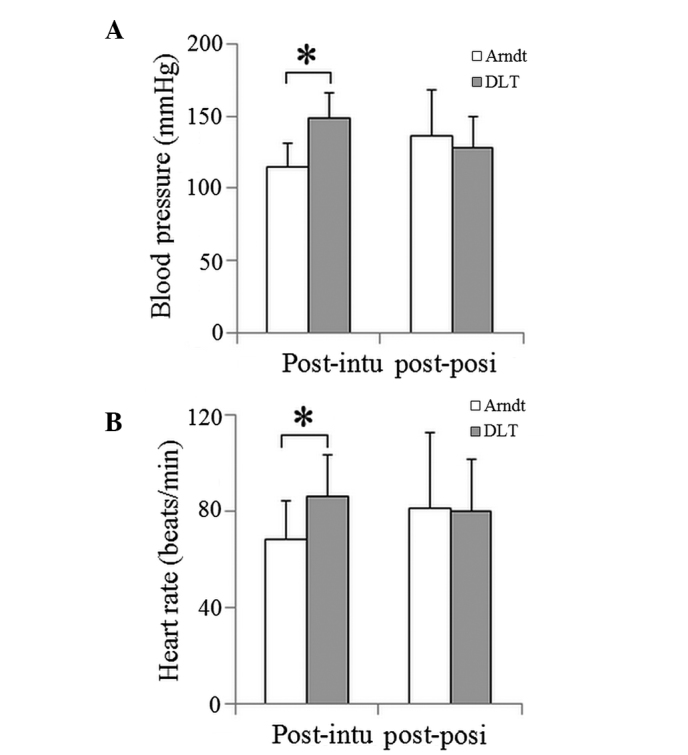
Hemodynamic changes of the two groups. (A) Blood pressure measured following intubation (Post-intu) and patient positioning (Post-posi). (B) Heart rate measured following intubation and patient positioning. Significant differences between group Arndt and group DLT were noted in blood pressure and heart rate following intubation (^*^P<0.05). DLT, double-lumen tube.

Significantly attenuated cardiovascular responses following the insertion of the LMA and endobronchial blocker or DLT were observed in group Arndt compared with group DLT (P<0.05) (Fig. 1). The cardiovascular responses induced by repositioning of the endobronchial blocker or the DLT were similar in the two groups. The respiratory parameters were comparable between the two groups with the exception of the peak airway pressure 5 min after OLV (Table IV). No significant differences were identified in oxygenation condition between the two groups (Table V).

**Figure 2:**
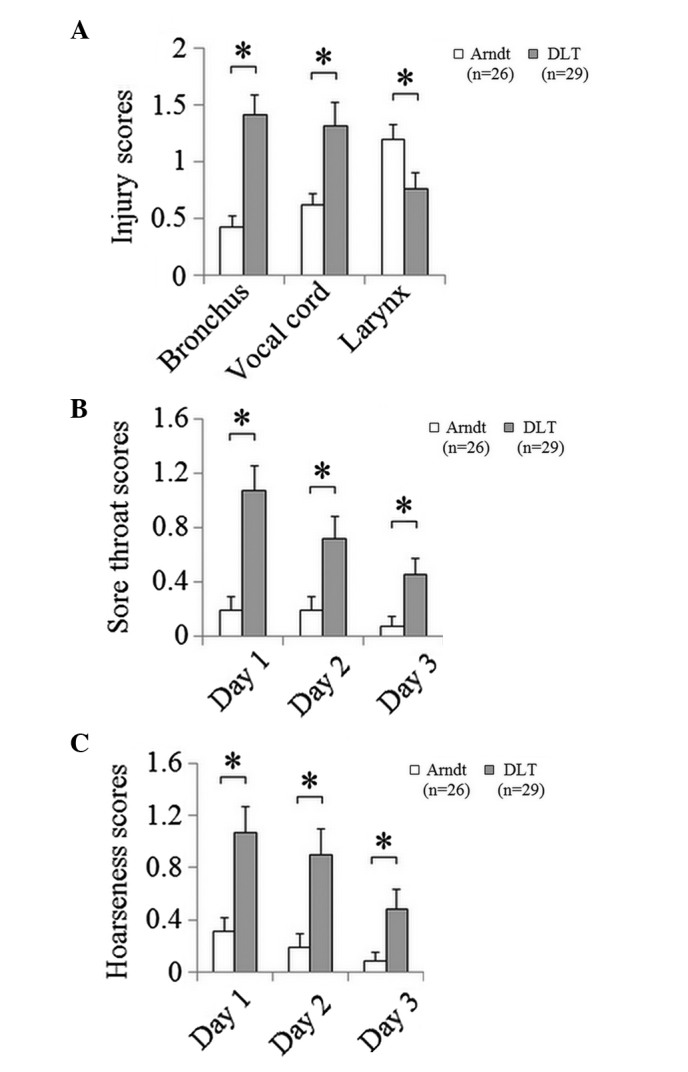
Post-operative airway injuries, sore throat and hoarseness. (A) The airway injuries were determined by bronchoscopy immediately subsequent to extubation and prior to patients’ awakening. The injury scores in the bronchus and vocal cords were significantly lower in the Arndt group, whereas the scores in the larynx were significantly lower in the DLT group. (B and C) Post-operative airway morbidity was obtained on the first three days after the surgery via the post-operative questionnaire. The incidences of sore throat and hoarseness were significantly lower in the Arndt group compared with those in the DLT group (^*^P<0.05). DLT, double-lumen tube.

Bronchoscopic examination revealed that the injury scores of the bronchus and vocal cords were significantly higher in group DLT than those in group Arndt, while the larynx injury score was lower in group DLT (Fig. 2A). The incidences of post-operative (three days after surgery) sore throat and hoarseness were significantly lower in group Arndt than those in group DLT (Fig. 2B and C).

## Discussion

DLTs are the most widely used devices for OLV; however, they have been reported to be associated with potential bronchus injury and sore throat (10). In cases of difficult airways, the intubation process of DLT may be further complicated when switching from a single-lumen tube with the help of the tube exchanger. The American Society of Anesthesiologists and the European Resuscitation Council have published algorithms naming the LMA as a primary option for the management of difficult and failed airways (11–13). Enabling OLV to be performed with the LMA would mean that difficult airways could be easily handled. However, the LMA alone is not able to provide OLV for thoracic surgeries. The Arndt endobronchial blocker has been demonstrated to be a useful technique that produces comparable surgical exposure in thoracotomy when combined with a conventional single-lumen tube. The present study evaluated the possibility of the combined use of the LMA and Arndt endobronchial blocker as an alternative OLV strategy in thoracic surgeries.

In this study, it was revealed that the combination of the LMA and the Arndt endobronchial blocker was able to provide effective surgical exposure via OLV, and additionally was associated with reduced fluctuations in hemodynamic response (Fig. 1), fewer airway injuries and less post-operative sore throat and hoarseness (Fig. 2).

The instant surge of blood pressure or heart rate of patients receiving DLTs may be attributed to the sudden contact of the tube tip with the bronchial wall during intubation. The increase may be more extreme in patients with chronic hypertension, which is a common pre-existing physical condition in patients undergoing thoracotomy. Episodes of intra-operative hypertension or hypotension and tachycardia may predispose the patient to adverse post-operative neurological or cardiac outcomes, including increased risk of stroke or myocardial infarction. Maintaining maximum hemodynamic stability is one of the principle goals of anesthetic management. However, hemodynamic fluctuations are difficult to avoid during or following direct laryngoscopy and tracheal intubation. The LMA has particular appeal in such cases. The use of the LMA was reported to result in an attenuated cardiovascular response compared with that found with direct laryngoscopy and endotracheal intubation (14). In the present study, it was shown that the hemodynamic responses of group Arndt during LMA and endobronchial blocker insertion were less fluctuated than those of DLT intubation.

The results of the present study demonstrated that, with the exception of injuries to the larynx, post-operative airway injuries were significantly lower in group Arndt than group DLT. Incidences of sore throat and hoarseness were also notably lower in group Arndt than group DLT. These findings were consistent with those of Knoll *et al* (10), who compared the airway injury caused by DLT or the Arndt endobronchial blocker. The LMA ProSeal may cause laryngopharyngeal mucosal injury not only by the intensity of the LMA cuff pressure but also in a time-dependent manner. A histological study demonstrated that prolonged use of the LMA ProSeal in the pig for <9 h was associated with no or mild alterations in the laryngopharyngeal mucosa, whereas clear signs of mucosal injury were observed after ≥12 h use (15). In the present study, due to the fact that the cuff pressure of the LMA was monitored and controlled by manometry, the higher risk of larynx injuries in group Arndt may have been caused by the prolonged use of the LMA due to the surgical time. Therefore, surgeries of long duration may not be recommended for the combination of the LMA and endobronchial blocker. However, for those less invasive day-case thoracic surgeries spreading rapidly throughout the world, this method may exhibit its advantages over DLTs.

Although not quantified, reduced secretions were observed in this study. Using this novel combination, it remains possible to perform tracheal suctioning through the internal channel of the FOB. In the present study, the FOB was advanced through the ventilation port of the LMA, the vocal cords and then to the trachea and bronchus. The process is relatively complicated, but suctioning can be performed under direct visualization. However, there was seldom requirement for suctioning in patients in the LMA group.

Complications resulting from the use of LMAs are known to be rare and were only present in 0.15% of >11,000 patients of all ages over a two-year period in a previous survey (16). When combined with the Arndt endobronchial blocker to achieve OLV, there may be concerns over the high pulmonary inflation pressures due either to increased airway resistance or to low lung compliance during OLV, which may lead to inadequate ventilation and gastric distension. It was reported in a previous study that the oropharyngeal leak pressure of the LMA ProSeal was 32 cmH_2_O (range, 12–40 cmH_2_O) (17). In the present study, the peak airway pressure during OLV was 24.7±4.8 cmH_2_O in the Arndt group, which is far below the leak pressure of the LMA ProSeal. Certain patients, even with body weight <90 kg, may have an inadequate seal with a size 4 LMA, and would require a change to a size 5 LMA. This has been supported by a number of previous reports (18,19). Following the correction of the inadequate seal of the LMA at the beginning of anesthesia, the OLV process could be performed without further complications.

The intubation parameters did not demonstrate any significant difference between the two groups in the present study. All the anesthesiologists involved in this study were more familiar with the DLT technique, despite being trained in the combination technique for several cases previously. It can be predicted that once the combination technique is completely mastered, fewer difficulties may be experienced during the airway establishing process of thoracic anesthesia. However, further studies in this respect are warranted.

In conclusion, the combination of the LMA and the Arndt endobronchial blocker can facilitate airway establishment, even for patients with difficult airways, in addition to causing fewer hemodynamic fluctuations in the intubation process and leading to lower incidences of post-operative sore throat and hoarseness. With these advantages, this novel combination is likely to serve as an effective alternative OLV strategy for thoracic surgeries, particularly for those minimally invasive day-case surgeries that are gaining increasing attention throughout the world.

## Figures and Tables

**Table I:** Demographic data.

Variable	Group Arndt, n=26	Group DLT, n=29
Age (years)^a^	55±15	57±13
Gender (M/F)	18/8	17/12
Height (cm)^a^	166±9	167±9
Weight (kg)^a^	62±11	64±10
FVC (%)^a^	83±18	89±13
FEV1 (%)^a^	89±25	89±14
Pre-op BP (mmHg)^a^	148±25	143±22
Pre-op HR (bpm)^a^	75±16	76±10
Smoking history (n)	16	14
Surgery duration (h)^a^	3.3±1.7	3.1±2.1
Anesthesia duration (h)^a^	3.7±1.9	4.2±1.8
OLV duration (h)^a^	1.7±1.2	1.8±1.1

a Values are presented as the mean ± standard deviation.

DLT, double-lumen tube; M/F, male to female ratio; FVC, forced vital capacity, percentage of predicted; FEV1, forced expiratory volume in the first second, percentage of predicted; pre-op BP, pre-operative blood pressure; pre-op HR, pre-operative heart rate; OLV, one-lung ventilation.

**Table II:** Distribution of types of surgical procedures.

Type of procedure	Group Arndt (n)	Group DLT (n)
VATS	7	6
Wedge resection	6	7
Lobectomy	5	7
Segmentectomy	2	2
Pneumonectomy	1	1
Mediastinal mass resection	3	2
Esophageal procedures	2	4

Group Arndt, n=26; Group DLT, n=29. No significant differences were identified between the two groups with regard to type of procedure. DLT, double-lumen tube; VATS, video-assisted thoracic surgery.

**Table III:** Airway parameters.

Parameter	Group Arndt	Group DLT	P-value
Mallampati grade	2.3±1.2	2.2±1.3	0.82
Cormack grade	2.5±1.4	2.3±1.3	0.72
Intubation attempts (n)	1.2±0.1	1.1±0.1	0.59
Intubation duration (min)	3.3±0.3	3.4±0.6	0.89
Positioning attempts (n)	1.2±0.1	1.2±0.1	0.88
Positioning duration (min)	5.2±0.7	3.0±0.4	0.08
Adjustments (n)	0.7±0.2	0.4±0.1	0.33

Values are presented as the mean ± standard deviation. Positioning, the positioning of the Arndt endobronchial blocker or DLT to facilitate one-lung ventilation; Adjustments, the adjustment of the Arndt endobronchial blocker or DLT due to inadequate lung isolation during surgery; DLT, double-lumen tube.

**Table IV:** Respiratory parameters of the two groups.

	PAP (mmHg)		Lung compliance (ml/cmH_2_O)	
		
Time	Group Arndt	Group DLT	Group Arndt	Group DLT
DLV 1	16.9±3.7	18.4±3.3	42.1±14.1	47.7±11.9
DLV 2	19.3±3.8	19.7±3.4	35.7±7.8	41.5±9.4
OLV 5	19.3±3.6^a^	19.7±3.6	35.7±7.8	41.5±9.5
OLV 20	24.6±4.8	25.0±3.9	25.7±9.8	27.8±7.6
OLV 60	24.7±4.9	25.5±3.8	25.7±8.9	25.8±6.7
DLV 5	24.9±4.3	26.3±3.3	26.0±8.8	26.3±7.4
DLV 10	20.4±4.9	19.9±4.7	43.0±17.8	42.4±13.7
DLV 20	22.2±5.2	21.1±4.5	37.6±17.7	41.2±12.8

Values are presented as the mean ± standard deviation.

a P<0.05 versus Group DLT.

DLV 1, 2, 5, 10 and 20 refer to the time-points at 1, 2, 5, 10 and 20 min of DLV, respectively; OLV 5, 20 and 60 refer to the time-points at 5, 20 and 60 min of OLV, respectively. PAP, peak airway pressure; DLT, double-lumen tube; DLV, double-lung ventilation; OLV, one-lung ventilation.

**Table V:** Oxygenation and lung isolation of the two groups.

Evaluation measure	Arndt	DLT	P-value
Pre-op PaO_2_/FiO_2_	400±52	362±62	0.15
OLV 20 PaO_2_/FiO_2_	209±89	184±82	0.29
DLV 20 PaO_2_/FiO_2_	461±60	454±65	0.65
Average exposure score	1.2±0.4	1.1±0.3	0.35
Average lung collapse score	1.2±0.6	1.1±0.4	0.54

Values are presented as the mean ± standard deviation. DLT, double-lumen tube; PaO_2_/FiO_2_, the ratio of the partial pressure of arterial oxygen to the fraction of inspired oxygen; Pre-op, pre-operative; OLV 20, at 20 min of one lung ventilation; DLV 20, at 20 min of double-lung ventilation following lung isolation.
